# Fistulectomy and primary sphincter reconstruction for high cryptoglandular anal fistula: a retrospective cohort study with long-term results

**DOI:** 10.1007/s00464-025-11585-9

**Published:** 2025-02-03

**Authors:** Karam Matlub Sørensen, Niels Qvist

**Affiliations:** 1 Research Unit of Surgery, Odense University Hospital, J.B. Winsløws, Vej 4, 5000 Odense C, Denmark; 2 Department of Clinical Research, University of Southern Denmark, Odense, Denmark

**Keywords:** Primary sphincter reconstruction, Anal fistula, Fistulectomy, Fecal incontinence, Quality of life

## Abstract

**Background:**

Surgical repair for high anal fistulas is challenging and can be associated with impaired functional outcomes. This study evaluated the long-term results of transsphincteric fistulectomy with primary sphincter repair for high anal fistulas in terms of recurrence, wound healing, fecal incontinence, and quality of life.

**Method:**

This retrospective cohort study included patients who underwent surgical repair for high anal fistulas between 2006 and 2015. Data were collected by reviewing patients’ electronic hospital records, including demographic characteristics, medical conditions, surgical findings, performed procedures, and follow-up data until the last recorded visit. Functional outcomes were assessed using self-reported online questionnaires for quality of life (RAND SF-36) and fecal incontinence (Wexner score).

**Results:**

Fifty-five patients were included. Primary healing was achieved in 42 (76%) patients, while 13 (24%) experienced recurrence. Following reoperations for recurrence, an additional 12 patients achieved healing, resulting in an overall healing rate of 98%. The median Wexner score was significantly higher in reoperated patients, and the median scores across all eight parameters of the RAND SF-36 were lower. None of the patients required proctectomy, and two ended with permanent stomas.

**Conclusion:**

Surgery for high anal fistulas is associated with a high success rate, but reoperations for recurrence are linked to considerable impairment in functional outcomes.

## Background

A high anal fistula is characterized by a fistula tract that traverses the upper two-thirds of the external sphincter muscle or above. High anal fistulas include high transsphincteric, suprasphincteric, and extrasphincteric anal fistulas [1–3]. Surgical procedures that preserve the sphincter complex are generally recommended to avoid impairing or losing continence function [1, 4]. Several different procedures have been described, but recurrence rates remain high, with no single treatment option demonstrating significant superiority in short- or long-term outcomes [5]. In 1961, Parks emphasized the importance of removing the entire fistula tract, particularly the internal orifice—the origin of the fistula from the infected gland—to prevent recurrence [6].

Fistulectomy and sphincter reconstruction (FSR) involves complete excision of the fistula tract tissue from the internal to the external orifice, including side branches, following the division of the overlying sphincter fibers. The sphincter is then repaired, and the overlying anal mucosa is reconstructed. This procedure can be performed as either a primary or staged operation. Although FSR reportedly has a high success rate [7–10], it has not been widely adopted, likely because of concerns about the potential risk of postoperative impairment of anal sphincter function [11]. However, long-term outcomes regarding functional results after FSR for complex anal fistulas remain to be investigated.

The aim of this study was to evaluate the outcome of FSR in the treatment of high anal fistulas of cryptoglandular origin. The primary objective was to assess the clinical recurrence of the fistula within a 2-year period following surgical treatment. The secondary objectives were the rate of wound healing (defined as epithelialization or scar formation), the need for stoma and proctectomy, and long-term outcomes related to fecal incontinence and quality of life. Quality of life was assessed using self-reported questionnaires, including the Wexner Fecal Incontinence Score and the RAND Short Form-36 (RAND SF-36) [12, 13].

## Method

This study was conducted as a retrospective cohort study of all patients who underwent transsphincteric fistula excision of a high anal fistula with primary anal sphincter reconstruction (FSR) at the Surgical Department of Odense University Hospital, Denmark, between 1 January 2006 and 31 December 2015. Our department serves as a tertiary referral center for complex anal fistulas in the Southern Region of Denmark, covering a population of approximately 1.2 million inhabitants. The study is reported in accordance with the STROBE guidelines [14].

Eligible patients were identified through electronic hospital records using a search strategy based on the World Health Organization’s International Classification of Diseases (ICD-10) code for anal fistula (DK60.3), combined with one or more of the following surgical procedure codes from the Nordic NOMESCO Classification of Surgical Procedures: excision of pathological tissue in the anal canal or perianal tissue (KJHA20), thermal destruction of pathological tissue in the anal canal or perianal tissue (KJHA30), suture of the anal sphincter (KJHC00), reconstruction of the anal sphincter (KJHC10), incision of anal fistula (KJHD20), excision of anal fistula (KJHD23), incomplete incision of anal fistula (KJHD30), complementary incision of anal fistula (KJHD33), other procedures in the anal canal (KJHW96), and other procedures in the rectum (KJHG96).

### Inclusion and exclusion

The inclusion criteria were living adults (≥ 18 years old) with perianal fistulas treated with FSR who consented to participate in a survey on functional outcomes and quality of life. The exclusion criteria were inflammatory bowel disease, rectovaginal fistulas, low anal fistulas, and fistulas treated solely with seton-sutures or other surgical procedures. Eligible patients were invited to participate in an online survey assessing functional outcomes through self-reported questionnaires, including the RAND SF-36 for quality of life [12] and the Wexner fecal incontinence score [13].

All surgical procedures were performed by dedicated proctologist surgeons. The anal fistulas were classified according to the classification established by Parks et al. [2], based on clinical findings during examination under general anesthesia and magnetic resonance imaging results. High anal fistulas [15], involving one-third or more of the sphincter, were treated with transsphincteric fistula excision followed by primary sphincter reconstruction, leaving the lateral part of the perianal skin incision open for drainage. All patients received intravenous cefuroxime and metronidazole preoperatively, which was continued for 3 days postoperatively. The patients were instructed to maintain wound hygiene through regular washing. Clinical follow-up was scheduled at the outpatient clinic 3 months after the surgical procedure and repeated as needed until fistula healing or treatment completion. Clinical or radiological suspicion of fistula recurrence was confirmed by examination under anesthesia.

### Data collection

Collected data included demographics (age, sex, height, weight, smoking and alcohol habits, and comorbidities), details of previous fistula surgery, preoperative stoma, seton drainage, perioperative findings (fistula characteristics and abscess), and clinical follow-up information. Follow-up data included recurrence, wound healing, postoperative fecal incontinence, postoperative stoma, reoperation, and proctectomy during the observation period, which extended from the index surgery to the last recorded visit. Data on functional outcomes at the time of the study were obtained through online self-reported questionnaires, including the RAND SF-36 and Wexner score. All data were entered into the REDCap electronic data-capture database, hosted by the Open Patient Data Explorative Network [16, 17]. This platform was used for obtaining consent to participate, administering the online self-completion questionnaires, and collecting raw data from the review of accessible electronic medical records. Only anonymized research data were stored and used for analysis.

### Statistical analysis

Continuous and categorical variables were analyzed using descriptive statistical tests, including the t-test, Wilcoxon rank-sum test, and Pearson correlation, as appropriate. Fistula recurrence was analyzed using logistic regression models (univariate and multivariate), testing the following variables for association as potential risk factors: age, sex, body mass index, smoking habit, alcohol consumption, health status, fistula duration, and fistula location. The relationship between fistula recurrence and time was assessed using survival models and the Kaplan–Meier method. A P-value of < 0.05 was considered statistically significant. All analyses were performed using STATA IC 16.1 software.

## Results

**Fig. 1:**
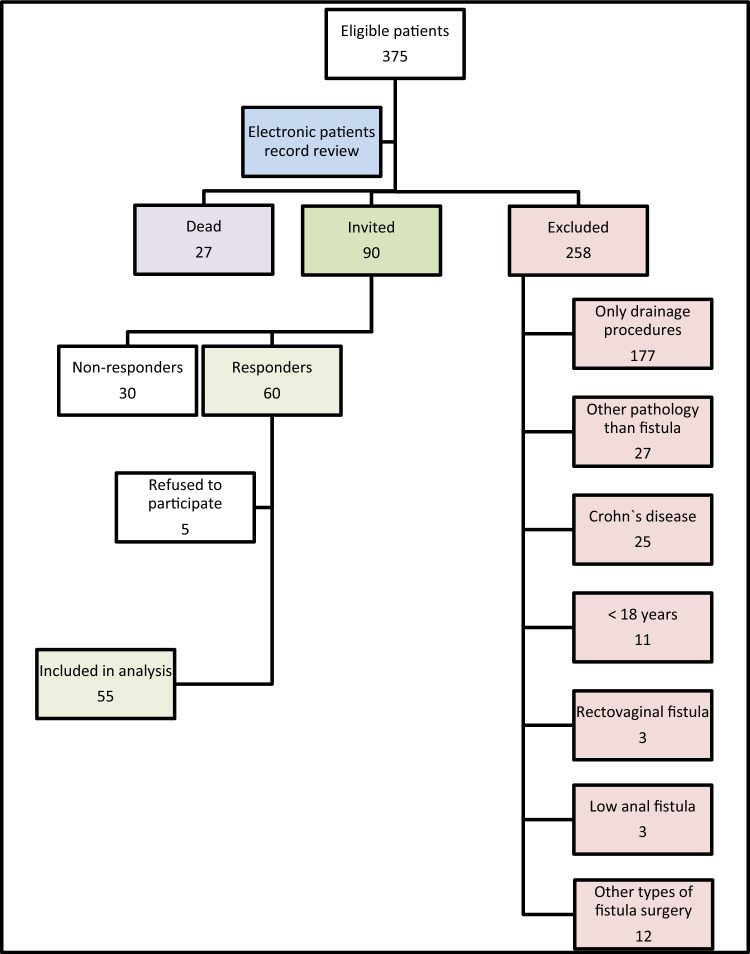
Study flowchart

Of 375 eligible patients, 258 were excluded (Fig. 1). Among these, 27 patients were deceased at the time of the study, and 90 patients were invited to participate. Of these, 30 did not respond and 5 declined participation, resulting in 55 patients included in the final analysis.

A complete dataset was available for all included patients regarding demographics, surgical findings, and follow-up data. Functional outcome survey data were available for all included patients, except for one patient with primary healing who had a missing Wexner score questionnaire.

The demographics of the included patients are summarized in Table 1. Nineteen patients (35%) were under the age of 40 years. Forty patients (73%) had no reported health issues, while the remaining 15 had one or more comorbidities. These included cardiovascular disease in 11 (20%) patients, pulmonary disease in 1 (2%), hepatic disease in 1 (2%), connective tissue disease in 1 (2%), and diabetes mellitus in 5 (9%). Most patients with comorbidities (93%) were 40 years of age or older.

**Table 1:** Demographic characteristics of the study population

Demographic variables (*n* = 55)	
Sex	
Male	26 (47%)
Female	29 (53%)
Age, years	45 (19–77)
BMI, kg/m^2^	28 (17–47)
Smoking habit	
Smoker	21(38%)
Quit	5 (9%)
Never Unknown	25 (46%) 4 (7%)
Alcohol consumption	
No consumption	11 (48%)
Within the recommended	10 (43%)
Above the recommended	2 (9%)
Health status	
Healthy	9 (39%)
Medical comorbidity	14 (61%)

Data are presented as n (%) or median (range). BMI, body mass index

The recommended alcohol consumption according to the Danish Health Council is 7 units/week for women and 14 units/week for men

Most patients (93%) had a history of anal abscess treated with surgical drainage, and 46 (84%) patients underwent seton drainage prior to surgery, with a median duration of 9 months (range: 2–30 months). Statistical analysis showed no significant relationship between recurrence and the duration of seton drainage.

Fifteen (27%) patients had previously undergone fistula surgery. The median duration of fistula symptoms was 12 months (range: 3–108 months). The fistula locations were almost equally distributed between anterior and posterior positions relative to an imaginary line traversing the anal opening, with no significant difference between the two sexes (P = 0.135). Most patients (n = 49; 89%) had a single-tract fistula. Twenty (36%) patients had a suprasphincteric type of anal fistula, while the remainder had high transsphincteric anal fistulas.

### Fistula recurrence and healing

**Fig. 2:**
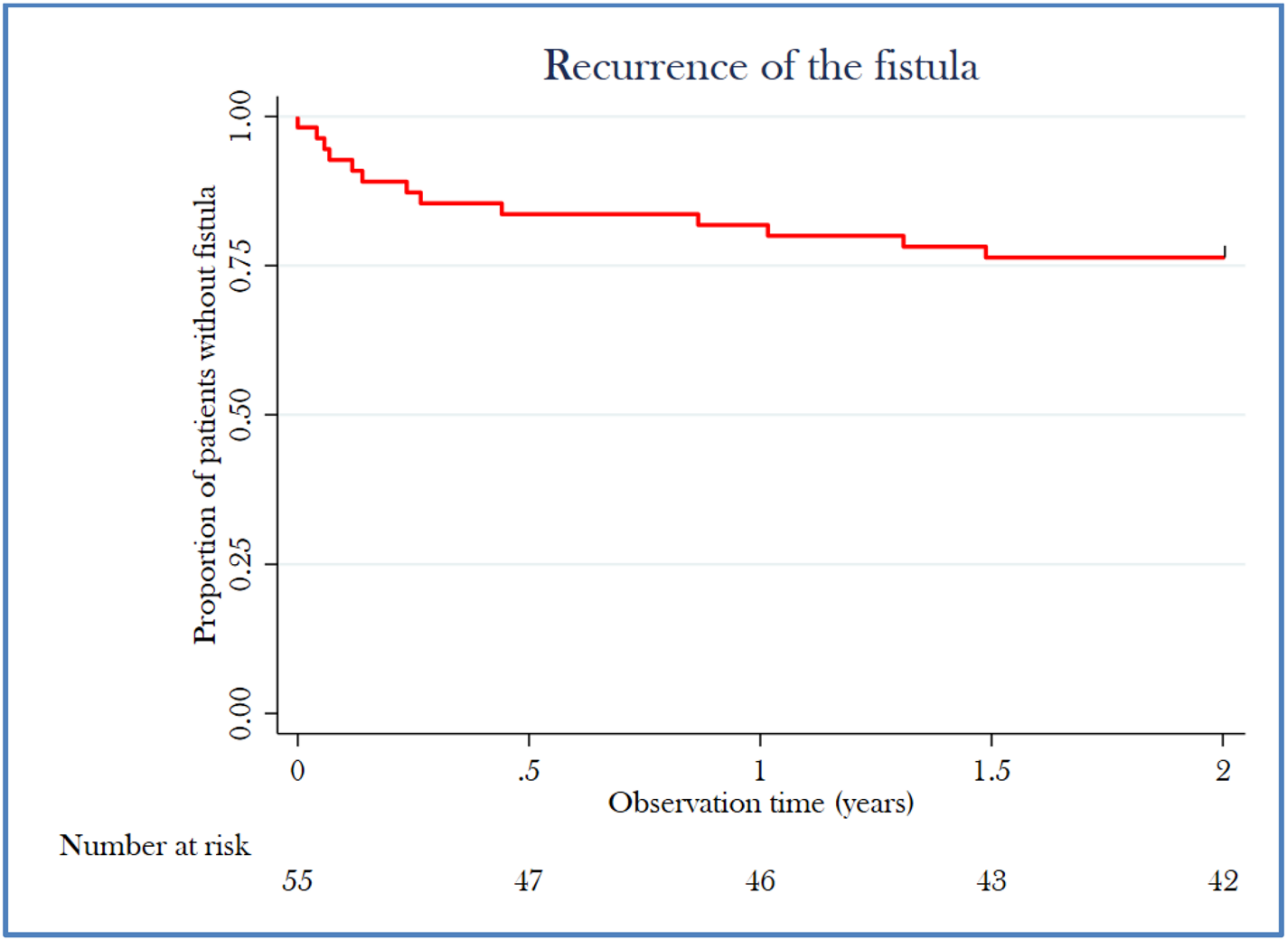
Kaplan–Meier estimate of fistula recurrence after primary fistulectomy and sphincter reconstruction

The median observation time (defined as the duration from surgery to the last visit) was 0.5 years (range: 0–5.5 years). Primary healing of the fistula was achieved in 42 (76%) patients, while 13 (24%) experienced recurrence. Of the 13 patients with recurrence, 12 underwent reoperation with repeated FSR. Among these, 8 patients underwent one reoperation, 3 required two reoperations, and 1 patient underwent three reoperations, ultimately achieving healing. This resulted in an overall healing rate of 98% after reoperation. One patient with recurrence deferred further surgery. Most recurrences occurred within the first 4 to 5 months after surgery (Fig. 2). Univariate and multivariate analyses using a logistic regression model did not identify any significant associations between the tested risk factors and fistula recurrence. Among the 15 patients with a history of previous fistula surgery, 11 (73%) achieved primary healing after FSR, and the remaining 4 achieved healing after repeated FSR.

One patient had a stoma preoperatively, which was reversed after successful fistula treatment with FSR. Four patients required a postoperative stoma due to recurrence; of these, two had their stomas reversed following successful reoperation, while two (3%) opted for a permanent stoma due to personal preference. None of the treated patients underwent proctectomy.

### Functional outcomes

According to the medical records, 50 (90%) patients reported no symptoms of fecal incontinence preoperatively. Two (4%) patients reported flatus incontinence, and three (6%) reported both flatus and stool incontinence, although none required the use of diapers.

Postoperatively, 16 (29%) patients experienced some degree of fecal incontinence. Of these, 13 had no continence issues preoperatively, while 3 had reported preoperative problems. Twenty-eight (51%) patients did not experience any fecal incontinence postoperatively, and for 11 (20%) patients, no information on fecal continence was available. One patient reported an improvement in fecal continence following surgery.

### Quality of life and fecal incontinence online survey (long-term results)

The median time between completing the online questionnaires and the last registered visit was 4.8 years (range: 0.6–11 years), with no significant difference between reoperated patients and those with primary healing. The median Wexner score for the study population was 4 (range: 0–20). A significant difference was observed between patients with primary healing and those requiring reoperation (*P* = 0.0003). Patients with primary healing had a median Wexner score of 2 (range: 0–17), while those who underwent reoperation had a median score of 12 (range: 4–20).

When relating the results of the online self-reported Wexner score to postoperative fecal incontinence data from the medical records, the median Wexner score was 10 (range: 0–20) in patients with documented postoperative fecal incontinence, 3 (range: 0–20) in those without postoperative fecal incontinence, and 3 (range: 0–12) in those for whom postoperative fecal incontinence was not mentioned in the medical records.

The parameters of the RAND SF-36 questionnaire were generally acceptable for the study population but were significantly lower (indicating higher disability) in reoperated patients (Table 2).

**Table 2:** Summary of the results of the online questionnaire for fecal incontinence and quality of life using the validated Danish version of the Wexner fecal incontinence score and the RAND SF-36 questionnaire, respectively

Online questionnaire	Total	No reoperation	Reoperation	*P* value
Quality of life (RAND SF-36)				
Physical function score	90 (5–100)	90 (5–100)	60 (20–100)	**0.005**
Role limitation due to physical health score	100 (0–100)	100 (0–100)	25 (0–100)	**0.001**
Role limitation due to emotional problems score	100 (0–100)	100 (0–100)	83 (0–100)	0.07
Energy-fatigue score	65 (10–100)	80 (10–100)	37.5 (20–85)	**0.002**
Emotional well-being score	82 (20–100)	86 (20–100)	64 (32–100)	**0.049**
Social functioning score	100 (25–100)	100 (25–100)	62.5 (37.5–100)	**0.012**
Pain score	80 (0–100)	90 (22.5–100)	45 (0–100)	**0.002**
General health score	60 (15–100)	65 (15–100)	47.5 (20–80)	**0.015**
Fecal incontinence				
Wexner score	4 (0–20)	2 (0–17)	12 (4–20)	** < 0.001**

*P* value below 0.05 was considered statistically significant

## Discussion

In this study, the primary healing rate after FSR was 76%, increasing to 98% after reoperation. No proctectomy was performed, and only two patients required a stoma. Long-term follow-up revealed significant impairment in functional outcomes for patients who required reoperation, while outcomes were acceptable for those with primary healing. A review of the medical records showed recurrence of the fistula in 13 of 55 treated patients, although it did not provide any insight into the reasons for recurrence. Statistical analysis of demographic and surgical variables did not reveal any significant associations with fistula recurrence.

In 2018, Seyfried et al. reported the outcomes of FSR in the treatment of high anal fistula in 424 patients, with a primary healing rate of 88.2%, a secondary healing rate of 95.8%, and 23% experiencing continence problems post-procedure [9]. The study concluded that FSR was feasible, had a low recurrence rate, and emphasized that no other procedure demonstrated better results for high transsphincteric fistulas. In a systematic review, Ratto et al. reported an overall success rate of 93.2% after fistulotomy or FSR, with a low morbidity rate and 12.4% worsening of fecal continence [18], advocating for well-designed studies to support FSR’s inclusion in treatment options for complex anal fistulas. Few studies have compared FSR with other techniques, with conflicting results. A randomized trial of an endoanal advancement flap versus FSR showed similar recurrence rates and continence outcomes [19], while a cohort study of 146 patients reported a lower recurrence rate and better functional outcomes with FSR than with an endoanal advancement flap [20]. In 2021, we reported a randomized trial comparing FSR and video-assisted anal fistula treatment, finding lower recurrence rates, better quality of life, and improved anal manometry with FSR [21]. A 2023 randomized trial comparing FSR with modified LIFT showed significantly lower recurrence rates with FSR but a higher incidence of postoperative flatus incontinence [22]. A recent systematic review confirmed FSR as a safe and effective procedure with pooled healing rates of 89%, sphincter dehiscence in 2% of cases, continence disturbances in 16%, and worsening continence in 8% [23]. However, significant heterogeneity in reported data leaves outcomes in high anal fistulas uncertain.

The results of this study indicate that patients who achieved primary healing with FSR had a low Wexner score and acceptable SF-36 questionnaire parameters, with a median time from study conduction to the last registered visit of approximately 5 years. This is likely the major finding of the study because it confirms the long-term efficacy of this technique. Approximately three-quarters of the patients achieved primary healing after a single FSR, with a relatively short treatment course of only a few months. However, recurrence of the fistula and the need for reoperation were significantly associated with impaired fecal continence and a negative impact on quality of life.

This study has several limitations. It was conducted as a single-center retrospective study without a control group or internal and external validation. Thirty eligible patients did not respond to the invitation to participate and five others declined, potentially introducing selection bias and overrepresenting patients with less favorable outcomes. Additionally, radiological evaluation of healing was not included in this cohort.

## Conclusion

FSR can be offered as an effective treatment option for patients with high anal fistula, providing favorable long-term functional outcomes. However, patients should be informed about the risks of recurrence and impairment of fecal continence.

## Data Availability

The research data, stored in a REDCap database, is not publicly available. However, a copy of the anonymized dataset and statistical analysis can be provided upon request to the corresponding author.

## References

[1] Steele SR, Kumar R, Feingold DL, Rafferty JL, Buie WD, Standards Practice Task Force of the American Society of Colon and Rectal Surgeons (2011) Practice parameters for the management of perianal abscess and fistula-in-ano. Dis Colon Rectum 54:1465–1474 22067173 10.1097/DCR.0b013e31823122b3

[2] Parks AG, Gordon PH, Hardcastle JD (1976) A classification of fistula-in-ano. Br J Surg 63:1–12 1267867 10.1002/bjs.1800630102

[3] Parks AG, Stitz RW (1976) The treatment of high fistula-in-ano. Dis Colon Rectum 19:487–499 964106 10.1007/BF02590941

[4] Bubbers EJ, Cologne KG (2016) Management of complex anal fistulas. Clin Colon Rectal Surg 29:43–49 26929751 10.1055/s-0035-1570392 PMC4755767

[5] Bhat S, Xu W, Varghese C, Dubey N, Wells CI, Harmston C, O’Grady G, Bissett IP, Lin AY (2023) Efficacy of different surgical treatments for management of anal fistula: a network meta-analysis. Tech Coloproctol 27:827–845 37460830 10.1007/s10151-023-02845-8 PMC10485107

[6] Parks AG (1961) Pathogenesis and treatment of fistuila-in-ano. Br Med J 1:463–469 13732880 10.1136/bmj.1.5224.463 PMC1953161

[7] Farag AFA, Elbarmelgi MY, Mostafa M, Mashhour AN (2019) One stage fistulectomy for high anal fistula with reconstruction of anal sphincter without fecal diversion. Asian J Surg 42:792–796 30738718 10.1016/j.asjsur.2018.12.005

[8] Roig GA, Jordan, Alos, Solana (1999) Immediate reconstruction of the anal sphincter after fistulectomy in the management of complex anal fistulas. Colorectal Dis 1:137–140 23577759 10.1046/j.1463-1318.1999.00021.x

[9] Seyfried S, Bussen D, Joos A, Galata C, Weiss C, Herold A (2018) Fistulectomy with primary sphincter reconstruction. Int J Colorectal Dis 33:911–918 29651553 10.1007/s00384-018-3042-6

[10] Ratto C, Litta F, Parello A, Zaccone G, Donisi L, De Simone V (2013) Fistulotomy with end-to-end primary sphincteroplasty for anal fistula: results from a prospective study. Dis Colon Rectum 56:226–233 23303152 10.1097/DCR.0b013e31827aab72

[11] Ratto C, Grossi U, Litta F, Di Tanna GL, Parello A, De Simone V, Tozer P, Zimmerman DED, Maeda Y (2019) Contemporary surgical practice in the management of anal fistula: results from an international survey. Tech Coloproctol 23:729–741 31368010 10.1007/s10151-019-02051-5 PMC6736896

[12] Bjorner JB, Thunedborg K, Kristensen TS, Modvig J, Bech P (1998) The Danish SF-36 Health Survey: translation and preliminary validity studies. J Clin Epidemiol 51:991–999 9817117 10.1016/s0895-4356(98)00091-2

[13] Jorge JM, Wexner SD (1993) Etiology and management of fecal incontinence. Dis Colon Rectum 36:77–97 8416784 10.1007/BF02050307

[14] von Elm E, Altman DG, Egger M, Pocock SJ, Gotzsche PC, Vandenbroucke JP, Initiative S (2008) The Strengthening the Reporting of Observational Studies in Epidemiology (STROBE) statement: guidelines for reporting observational studies. J Clin Epidemiol 61:344–349 18313558 10.1016/j.jclinepi.2007.11.008

[15] Sandborn WJ, Fazio VW, Feagan BG, Hanauer SB, Committee AGACP (2003) AGA technical review on perianal Crohn’s disease. Gastroenterology 125:1508–1530 14598268 10.1016/j.gastro.2003.08.025

[16] Harris PA, Taylor R, Minor BL, Elliott V, Fernandez M, O’Neal L, McLeod L, Delacqua G, Delacqua F, Kirby J, Duda SN, R. EDCap Consortium (2019) The REDCap consortium: building an international community of software platform partners. J Biomed Inform 95:103208 31078660 10.1016/j.jbi.2019.103208 PMC7254481

[17] Harris PA, Taylor R, Thielke R, Payne J, Gonzalez N, Conde JG (2009) Research electronic data capture (REDCap)–a metadata-driven methodology and workflow process for providing translational research informatics support. J Biomed Inform 42:377–381 18929686 10.1016/j.jbi.2008.08.010 PMC2700030

[18] Ratto C, Litta F, Donisi L, Parello A (2015) Fistulotomy or fistulectomy and primary sphincteroplasty for anal fistula (FIPS): a systematic review. Tech Coloproctol 19:391–400 26062740 10.1007/s10151-015-1323-4

[19] Perez F, Arroyo A, Serrano P, Sanchez A, Candela F, Perez MT, Calpena R (2006) Randomized clinical and manometric study of advancement flap versus fistulotomy with sphincter reconstruction in the management of complex fistula-in-ano. Am J Surg 192:34–40 16769272 10.1016/j.amjsurg.2006.01.028

[20] Roig JV, Garcia-Armengol J, Jordan JC, Moro D, Garcia-Granero E, Alos R (2010) Fistulectomy and sphincteric reconstruction for complex cryptoglandular fistulas. Colorectal Dis 12:e145–e152 19604292 10.1111/j.1463-1318.2009.02002.x

[21] Sorensen KM, Moller S, Qvist N (2021) Video-assisted anal fistula treatment versus fistulectomy and sphincter repair in the treatment of high cryptoglandular anal fistula: a randomized clinical study. BJS Open 5:zrab097 34611700 10.1093/bjsopen/zrab097 PMC8493008

[22] Awad PBA, Hassan BHA, Awad KBA, Elkomos BE, Nada MAM (2023) A comparative study between high ligation of the inter-sphincteric fistula tract via lateral approach versus fistulotomy and primary sphincteroplasty in high trans-sphincteric fistula-in-ano: a randomized clinical trial. BMC Surg 23:224 37559044 10.1186/s12893-023-02117-0 PMC10413541

[23] Iqbal N, Dilke SM, Geldof J, Sahnan K, Adegbola S, Bassett P, Tozer P (2021) Is fistulotomy with immediate sphincter reconstruction (FISR) a sphincter preserving procedure for high anal fistula? A systematic review and meta-analysis. Colorectal Dis 23:3073–3089 34623747 10.1111/codi.15945

